# Drug-Induced- or Rheumatic- Valvular Heart Disease in Patients Exposed to Benfluorex?

**DOI:** 10.1371/journal.pone.0160011

**Published:** 2016-08-03

**Authors:** Florent Le Ven, Zarrin Alavi, Yannick Jobic, Yves Etienne, Romain Didier, Raphaël Porcher

**Affiliations:** 1 Service de Cardiologie, Hôpital de la Cavale Blanche CHRU Brest, France; 2 Université Européenne de Bretagne, Université de Brest, INSERM CIC 1412, CHRU Brest, France; 3 Assistance Publique Hôpitaux de Paris, Hotel-Dieu, Centre d’Epidémiologie Clinique, Université Paris Descartes, Inserm U1153, Paris, France; Hospital Universitario LA FE, SPAIN

## Abstract

There is a risk of misdiagnosis between benfluorex-induced VHD and acute rheumatic fever (ARF)-related VHD due to common characteristics of both etiologies. We aimed at estimating the probability for a patient exposed to benfluorex presenting with VHD to have, at the same time, a history of ARF-related VHD. Such epidemiological approach could help at reducing the risk of misdiagnosis. We used INSEE data and related literature as well as various modeling hypotheses to drive and test a formula for calculating the probability of a patient presenting with VHD and a history of benfluorex intake to have a prior history of ARF-related VHD. Different scenarios were estimated by a Markov model on the life course of people born in France between 1940 and 1960. Sensitivity analyses were performed under these scenarios. According to the different scenarios and gender, the probability that a patient born between 1940 and 1960 presenting with VHD and a history of benfluorex intake would have had a prior history of ARF-related VHD varied from 0.2% to 2.7%. The probabilities by the year of birth were as follows: 0.8%–2.7% for a patient born in 1940, < 0.5% in all scenarios for patients born after 1955, and < 0.2% in all scenarios for patients, born in 1960. Our results indicate that the burden of ARF-related VHD is low in the patient population exposed to benfluorex. The probability of ARF related VHD should not be over-estimated in the diagnostic procedure of VHD.

## Introduction

Anorexigen drugs derived from fenfluramine (dexfenfluramine) were marketed as early as 1960’s worldwide and banned from marketing in 1997. Nevertheless, fenfluramine-derived drug, i.e. benfluorex, remained on some markets around the world as antidiabetics medication. It was then withdrawn from Italy and Spain in 2004 and 2005, respectively. All these withdrawals were done following reports of clinical evidence of drug-induced (DI) valvular heart disease (VHD) and pulmonary arterial hypertension. From 2006 until 2009, benfluorex-containing drugs were available only in Cyprus, Portugal and France after the drug’s marketing authorizations were revoked throughout Europe.

The European Medicines Agency recommended the withdrawal of all medicines containing benfluorex in the European Union in December 2009.

Following the withdrawal of benfluorex, French government recommended screening for VHD by echocardiography in the benfluorex exposed population (e.g. 2012 mean age of 63.6 ± 10.8 [1]). Benfluorex-induced VHD and acute rheumatic fever (ARF)-related VHD share some common macroscopic and histological characteristics. However, there are more specific features distinguished for each etiology that can help at detection of one specific etiology over the other. Since there is a risk of diagnostic confusion between benfluorex-induced VHD and ARF-related VHD due to common characteristics of both etiologies of VHD, estimating the number of people born between 1940 and 1960 with ARF- related VHD and still alive in 2012 becomes relevant. Given this estimation, we aimed at calculating the probability for a patient exposed to benfluorex presenting with VHD at the same time having a history of ARF-related VHD. In this manner, we can provide the health care team with substantial data for a better and more accurate diagnosis of VHD thus reducing the risk of confusion in regard to VHD etiology, i.e. between DI disease or complication of previous ARF.

## Methods

The main objective of our work was to estimate the probability of a patient presenting with VHD and a history of benfluorex intake to have a prior history of ARF-related VHD. We first derived an equation and then estimated the relevant number of patients in the equation by modeling the evolution of the cohort of people born in France between 1940 and 1960 using a Markov model. The model was populated by using data for the birth and mortality rates in France, as well as the literature, and sensitivity analyses were carried out under different scenarios.

### Data sources

The following data were collected from INSEE (French National Institute of Statistics and Economic Studies) web site (http://www.insee.fr):

- Live birth registries between 1940 and 1960,
- Infants’ deaths registries since 1940,
- Mortality rates by age between 1946 and 2012

Valvular heart disease (VHD)-related excess mortality rates were obtained from the literature [2]. The risk of benfluorex-induced VHD was derived from the results of the works of Tribouilloy et al [1, 3].

### Assumptions and parameter values

**Fig 1:**
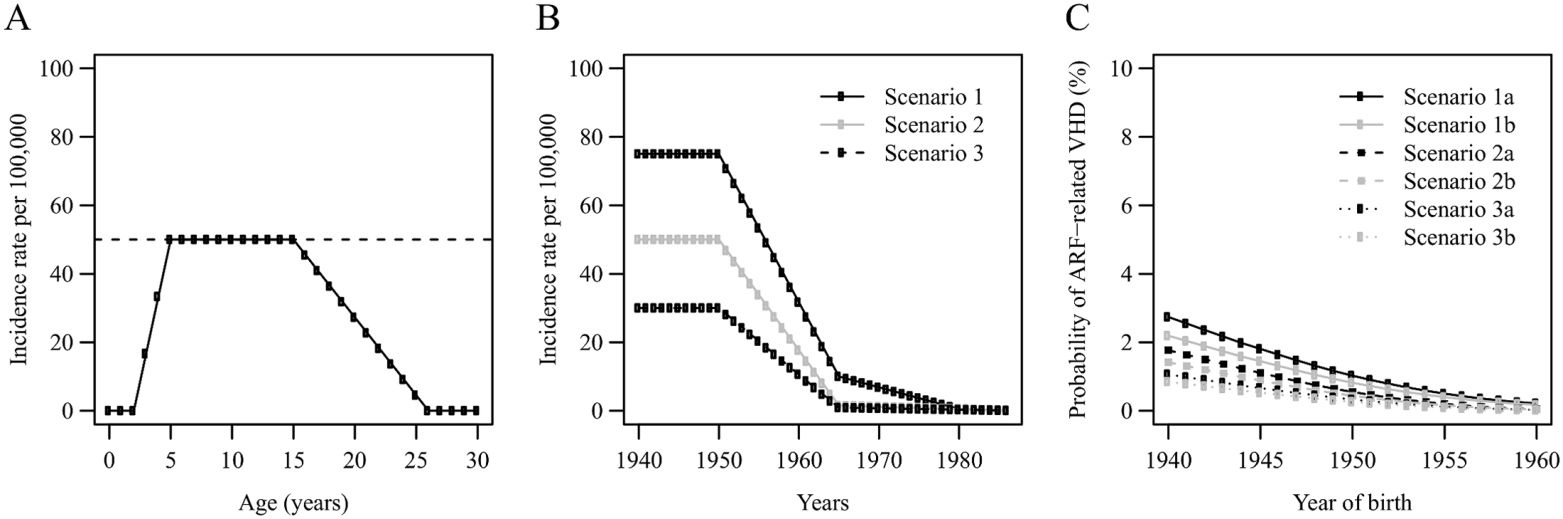
ARF incidence rates and resulting probability of ARF-related VHD. **Panel A** displays the shape of the incidence rate of ARF according to patient age between 0 and 30 years, for a reference incidence rate of 50 per 100,000. **Panel B** presents the reference incidence rate for each year between 1940 and 1986 for the three core scenarios. The results on the probability of ARF-related VHD in a patient presenting in 2013 with VHD and a history of benfluorex are given on the **panel C**, according to the year of birth of the patient.

Our modeling relies on the following assumptions:

1. Benfluorex intake has been independent of any occurrence of acute rheumatic fever (ARF) and any ARF-related VHD.
2. The probability to develop benfluorex-induced VHD was the same between patients without previous occurrence of ARF and patients with previous occurrence of ARF among patients who did not develop ARF-induced VHD. Patients with ARF-induced VHD could not additionally develop benfluorex-induced VHD (it is worth mentioning that this could happen in real life).
3. Age-specific mortality rates after 1 year of age have been steady between 1941 and 1946 (because detailed mortality rates were not available for the 1941 to 1945 period).
4. ARF affected children between 3 to 25 years old with an incidence rate estimated to be constant between 5 and 15 years (reference incidence rate thereafter) but linearly increasing between 3 and 5 and linearly decreasing between 15 and 25 years (Fig 1A).
5. ARF patients could develop a VHD which could cause an excess mortality as early as the age of 20. In the absence of VHD, ARF patients’ mortality was the same as that of the general population.
6. ARF affected equally male and female children.
7. The proportion of patients with ARF-related VHD was the same in the French general population born in France and abroad between 1940 and 1960 as in the population born in France between 1940 and 1960.

For ARF patients with VHD, the hazard ratio of death has been estimated at 1.75 (95% C.I. 1.61 to 1.90) in a community-based study, and 1.36 (1.15 to 1.62) when pooling population-based studies [2]. Since a value between 1.61 and 1.62 belonged to both confidence intervals, we chose in our study a conservative estimation of 1.5. The probability of echo-based VHD in patients treated with benfluorex was set at 16%. We considered for this figure the difference in the overall frequency of left heart valve regurgitation in patients exposed to benfluorex compared with controls in the study by Tribouilloy et al [1]. We additionally varied this parameter, ranging from 7% to 22%, the upper limit being the upper 95% confidence limit of the estimate in reference 1 and the lower limit being the point estimate of the probability of echocardiography features of DI-VHD in patients previously exposed to benfluorex [3].

### Conditional probability of ARF-related VHD

We aimed at calculating the probability that a patient presenting with VHD and a history of benfluorex intake would have a prior history of ARF-related VHD. Under assumptions 1 and 2 above, this probability can be expressed as the ratio of two probabilities:

P(ARF−induced VHD | VHD and benfluorex intake) =P(ARF−induced VHD) /P(VHD | benfluorex intake)

Where *P*(ARF-induced VHD) denotes the probability of ARF-induced VHD in the population under study. To the best of our knowledge, there are no epidemiological data allowing to estimate this probability for the population of interest. We therefore relied on a Markov model to provide estimates of this probability under different scenarios.

### Markov model and scenarios

Markov modeling was first used to reconstruct the number of patients born between 1940 and 1960 alive each year from 1940 until 2013. The model used one-year intervals, and the analysis was performed separately for females and males, since both have markedly different mortality rates. Patients entered the cohort according to gender-specific numbers of live births between 1940 and 1960. Age- and gender-specific mortality rates until 2012 were then used to calculate the survivors of each age at each time interval. This allowed building the cohort population according to age and gender for each year between 1940 and 2013. Age-specific ARF incidence rates were used to derive the number of patients who developed ARF in a given year. Then, for each year of birth and gender, the number of patients who developed ARF-related VHD in a given year was computed by presuming that a fixed fraction of patients with ARF would develop VHD (see scenarios below). Increased mortality rate assumption for these patients after the age of 20 was then applied to obtain the number of survivors for each remaining year until 2013. Given the relatively small number of patients with ARF-related VHD as compared to the whole population, we presumed that the increased mortality rate did not affect the cohort of patients without ARF-related VHD. The probability *P*(ARF-induced VHD) in 2013 was then computed as the ratio of the number of patients with ARF-related VHD estimated to be still alive in 2013 to the total number of patients of the cohort still alive in 2013. This probability, i.e. *P*(ARF-induced VHD) in 2013, could be computed on the entire population, or stratified by gender and age (i.e. by year of birth).

Several scenarios were used to provide a reasonable range of estimations. For ARF incidence over the course of time, three scenarios for the reference incidence rate were built (Fig 1B):

- Scenario 1 was built based on the incidence rate reported in Denmark between 1940 and 1970 [4].
- Scenario 2 was built using the upper bound of the incidence rate reported in England in the early 1950’s [5] with a similar shape as the first scenario, but with a virtually null incidence as early as 1965.
- Scenario 3 was built using the lower bound of the incidence rate reported in England in the early 1950’s [5] with a similar shape as in scenario 2.

Then, for each of the above scenarios, it was hypothesized that either 50% (sub-scenarios denoted by the letter a) or 40% (b) of ARF patients would develop VHD. This brought forward six scenarios for calculating the number of patients with ARF-related VHD. These scenarios—in particular (1) and (a)–can be regarded as excess scenarios that are likely to over-estimate the number of patients with ARF-related VHD.

## Results

Table 1 displays the number of people born between 1940 and 1960 with ARF in accordance with the above described scenarios (1, 2 and 3).

**Table 1:** Estimated number of patients developing ARF among those born in France between 1940 and 1960 under the different scenarios.

Scenario	All	Men	Women
Scenario 1	67863	34298	33565
Scenario 2	37828	19112	18716
Scenario 3	22697	11467	11229

According to the different scenarios and gender, the probability that a patient born between 1940 and 1960 presenting with VHD and a history of benfluorex intake would have had a prior history of ARF-related VHD varied from about 0.2% to 2.7% (Table 2).

**Table 2:** Estimated probability of a history of ARF-related VHD in a patient born between 1940 and 1960 presenting with VHD and a history of benfluorex intake under the different scenarios. Results are expressed as percent. Estimates are obtained with a probability of VHD in patients treated by benfluorex of 16%, whereas the range obtained with probabilities varying from 7% to 22% are given in parentheses.

Scenario	All	Men	Women
Scenario 1a	1.04 (0.76;2.38)	0.91 (0.66;2.08)	1.16 (0.85;2.66)
Scenario 1b	0.83 (0.61;1.90)	0.73 (0.53;1.66)	0.93 (0.68;2.12)
Scenario 2a	0.58 (0.42;1.33)	0.51 (0.37;1.16)	0.65 (0.47;1.48)
Scenario 2b	0.46 (0.34;1.06)	0.40 (0.29;0.93)	0.52 (0.38;1.18)
Scenario 3a	0.35 (0.25;0.80)	0.30 (0.22;0.69)	0.39 (0.28;0.89)
Scenario 3b	0.28 (0.20;0.64)	0.24 (0.18;0.56)	0.31 (0.23;0.71)

When further looking at these probabilities by the year of birth, we estimated the following probabilities (Fig 1C):

- between 0.8% and 2.7% for a patient born in 1940,
- less than 0.5% in all scenarios for patients born in 1955 or after,
- less than 0.2% in all scenarios for patients born in 1960.

## Discussion

In this work, we aimed at estimating the probability for a patient exposed to benfluorex presenting with VHD at the same time having a history of ARF-related VHD.

Data including clinical case reports, case series, case-control studies [6], a large-scale cohort study [7] and randomized trial [8] have been accumulated leading to the consistent conclusion that benfluorex, a fenfluramine derivative, is associated with the development of cardiac valvular regurgitation. Benfluorex induces restrictive drug-induced valvular heart disease (DI-VHD). Activation of the 5-HT2_B_ receptor by norfenfluramine (the active metabolite of benfluorex) is thought to be the main mechanism of development of DI-VHD. 5-HT2_B_ receptors activation produces endocardial fibrosis of the leaflets with chordae tendinae fusion and retraction. These valvular alterations may at first glance mimic ARF-related VHD. However, the absence of symptoms during childhood and pregnancy or prior benfluorex exposure, as well as the paucity of valvular calcifications, and the slight increase in valvular thickness should lead to conclusive diagnosis of DI-VHD. Understanding heart valve macro- and micro-structures plays a key role in the diagnosis and treatment of VHD. Accordingly, the heart valve architecture is well preserved, i.e. without inflammation or neovascularisation in DI-VHD in contrast with ARF-related VHD.

Thanks to antibiotic therapy, the incidence of ARF-related VHD has declined in Western countries. Yet, other etiologies of VHD have increased, e.g. degenerative, infectious, systemic or congenital DI-VHD. Sharing some similar histological and pathological characteristics with ARF-VHD, DI-VHD has emerged since the 1960s when ergot alkaloids (initially methysergide [9], then ergotamine) were being used as migraine prophylaxis and anorexigens as diet pills. Of note, dexfenfluramine and benfluorex were prescribed to at least 10 million patients in France [10]. Given the similar morphological features of both etiologies as well as the previous epidemiological evidence, it could be a challenge to distinguish between ARF- and DI-VHD. Echocardiography has been the gold standard to detect VHD and evaluate its etiology and severity. However, there is rarely a baseline pre-drug exposure echocardiography available. Thus VHD diagnosis is essentially based on the study of valve texture and kinetics and the analysis of the subvalvular apparatus (for the mitral valve). Both aortic and mitral valves can be affected. Aortic regurgitation (AR) rises from abnormalities of the aortic leaflets, their supporting structures in the aortic root and annulus, or both [11]. AR may rise from abnormalities of the aortic valves including congenital defects such as rheumatic heart disease with fusion of the commissures and retraction of the aortic valve leaflets due to scarring and fibrosis; myxomatous infiltration of the aortic valve; tumors; infective endocarditis; atherosclerotic degeneration; connective tissue disorders (e.g. Marfan syndrome); inflammatory diseases (e.g. aortitis; antiphospholipid syndrome); and the use of anorectic drugs or ergot-derived compounds [11]. Double mitral and aortic restrictive regurgitation in the absence of a history of rheumatic fever is highly suggestive of DI-VHD. Valvular thickening is typically observed but is not always present and is often minimal or moderate, with no severe calcification. Histological examination reveals dense fibrosis generally localised on the endocardial valve surface, with no alteration of valve architecture in contrast with ARF-related VHD. The latter induces more diffuse histological abnormalities of valvular tissue and an inflammatory infiltrate, which is usually not observed in drug-induced (DI) disease [12].

The overlap of shared features between both etiologies could most likely lead to an over-diagnosis of a rheumatic etiology historically more known [13]. Our data suggest that a patient with a history of benfluorex intake and a VHD has a < 3% likelihood of ARF related VHD. Tribouilloy et al. reported that patients with previous benfluorex exposure, i.e. at least 3 months, vs. matched unexposed controls showed a > 3-fold, > 5-fold, > 2-fold increased risk of aortic and/or mitral valve regurgitations, aortic regurgitation, and mitral regurgitation, respectively [1]. Noteworthy, the lower limit of 7% for the probability of echo-based VHD in patients treated with benfluorex, was based on the work of Tribouilloy et al. who found that echocardiography can detect features regarded as highly suggestive of DI-VHD in 6.8% of patients previously exposed to benfluorex. Such features are very uncommon (0.26%) in diabetic subjects not exposed to drugs known to induce VHD [3]. Weill et al. reported a 3-fold increased risk of hospital admission for mitral regurgitation and a 4-fold increased risk of hospitalization for aortic regurgitation and valvular replacement surgery during the first 2 years after exposure to benfluorex [7].

It is worth mentioning that similar increased valvular risks were reported for dexfenfluramine in the case control study by Khan et al [14].

Taken all together, clinicians should be cautious when facing patients exposed to dexfenfluramine, benfluorex and other anorexigens in diagnosis and management of VHD. Unfortunately relevant epidemiological data are lacking for ergotamine exposure.

The major limitation of this work is that it was based on modeling, which implies a simplification of the reality. Moreover, the model relied on several assumptions that are unverifiable, such as the same proportion of patients with ARF-related VHD in the French general population born in France and born abroad or the independence of occurrence of ARF or ARF-related VHD and benfluorex intake. In the absence of objective data, to date, there is no other possible way to parameterize the model than to make other unverifiable assumptions The latter were supported by reports on ARF epidemiology, such as a similar ARF incidence in both sexes [4, 15]. We also acknowledge the paucity of data concerning ARF epidemiology in France between 1940 and 1970. This was accounted for by using different scenarios for the incidence rate during the study period, derived from epidemiologic data from countries with similar levels of development (Denmark and England). We also made conservative assumptions, such as considering that any VHD in a patient with ARF-related VHD would be exclusively due to ARF and not to benfluorex intake. All our modeling choices were made explicit, so that the results could be reproduced under the same scenarios, or developed under other scenarios. To populate our study we however also used reliable data such as the number of live births and the age and gender-specific mortality rates obtained from the French National Institute of Statistics and Economic Studies (INSEE). Results were obtained under several scenarios and conclusions were robust to these scenarios, although the scenarios influenced the numerical values obtained. At least we expect to have covered a wide range of possible situations so that the estimate provided may encompass reasonable estimates of the probability of ARF-related VHD in patients exposed to benfluorex and presenting with VHD.

VHD includes several different pathophysiological mechanisms, presentations and natural histories [16]. The population has evolved and today’s VHD etiology has gone through a transformation over the last several decades. This transformation has emerged due to factors such as ARF eradication in industrial world, increased life span and emergence of degenerative, iatrogenic VHD [16]. Research works based on modeling allow reconsidering the preconceived ideas in the light of epidemiological data. ARF-related VHD diagnosis is very often performed retrospectively, in patients who underwent valvular surgery but did not have a pathological analysis done. This diagnosis is often based on unreliable clinical evidence such as a history of angina, or amygdalectomy. In such a situation where there is no reliable and conclusive diagnostic test, one major driver of the predictive value remains the prevalence. And when the prevalence becomes very low, even a test with fair sensitivity and specificity has a low positive predictive value. Our original modeling approach can reveal that one etiology (infectious disease) has been set forth instead of a new and not well-known etiology (DI-disease) in diagnosis and management of VHD since 1940’s.

## Conclusion

Our results indicate that the burden of ARF-related VHD is low in the patient population exposed to benfluorex. The probability of ARF related VHD should not be over-estimated when facing a patient presenting with VHD.
